# Web-Based Survey Application to Collect Contextually Relevant Geographic Data With Exposure Times: Application Development and Feasibility Testing

**DOI:** 10.2196/publichealth.8581

**Published:** 2018-01-19

**Authors:** Abby Rudolph, Karin Tobin, Jonathan Rudolph, Carl Latkin

**Affiliations:** ^1^ Department of Epidemiology Boston University School of Public Health Boston, MA United States; ^2^ Department of Health, Behavior, and Society Johns Hopkins University Bloomberg School of Public Health Baltimore, MD United States; ^3^ Independent Consultant Warminster, PA United States

**Keywords:** spatial analysis, geographic mapping, substance-related disorder

## Abstract

**Background:**

Although studies that characterize the risk environment by linking contextual factors with individual-level data have advanced infectious disease and substance use research, there are opportunities to refine how we define relevant neighborhood exposures; this can in turn reduce the potential for exposure misclassification. For example, for those who do not inject at home, injection risk behaviors may be more influenced by the environment where they inject than where they live. Similarly, among those who spend more time away from home, a measure that accounts for different neighborhood exposures by weighting each unique location proportional to the percentage of time spent there may be more correlated with health behaviors than one’s residential environment.

**Objective:**

This study aimed to develop a Web-based application that interacts with Google Maps application program interfaces (APIs) to collect contextually relevant locations and the amount of time spent in each. Our analysis examined the extent of overlap across different location types and compared different approaches for classifying neighborhood exposure.

**Methods:**

Between May 2014 and March 2017, 547 participants enrolled in a Baltimore HIV care and prevention study completed an interviewer-administered Web-based survey that collected information about where participants were recruited, worked, lived, socialized, injected drugs, and spent most of their time. For each location, participants gave an address or intersection which they confirmed using Google Map and Street views. Geographic coordinates (and hours spent in each location) were joined to neighborhood indicators by Community Statistical Area (CSA). We computed a weighted exposure based on the proportion of time spent in each unique location. We compared neighborhood exposures based on each of the different location types with one another and the weighted exposure using analysis of variance with Bonferroni corrections to account for multiple comparisons.

**Results:**

Participants reported spending the most time at home, followed by the location where they injected drugs. Injection locations overlapped most frequently with locations where people reported socializing and living or sleeping. The least time was spent in the locations where participants reported earning money and being recruited for the study; these locations were also the least likely to overlap with other location types. We observed statistically significant differences in neighborhood exposures according to the approach used. Overall, people reported earning money in higher-income neighborhoods and being recruited for the study and injecting in neighborhoods with more violent crime, abandoned houses, and poverty.

**Conclusions:**

This analysis revealed statistically significant differences in neighborhood exposures when defined by different locations or weighted based on exposure time. Future analyses are needed to determine which exposure measures are most strongly associated with health and risk behaviors and to explore whether associations between individual-level behaviors and neighborhood exposures are modified by exposure times.

## Introduction

### Geographic Information Systems Approaches in Substance Use and Infectious Disease Research

The risk environment and geography both play important roles in shaping overdose risk, risk behaviors associated with the transmission of sexually transmitted infections including HIV and hepatitis C virus (HCV), and the use of prevention and treatment services [1-6]. For example, geographic data are increasingly used in HIV and HCV prevention research and substance use research to identify hot spots for diseases, poor health outcomes, and risk behaviors [7-10] and health service deserts (ie, areas with decreased availability of or access to health services) [11,12]. Geographic information systems approaches are also used to evaluate the association between proximity to health services and their use (eg, clinics [10,13], drug treatment programs [11,12,14], and syringe exchange programs [15-18]) or travel distance as a barrier [19-22] to their use. Furthermore, studies that aim to characterize the risk environment link contextual factors with individual-level data to better understand how the built and social environment influence individual-level behaviors and health outcomes [23].

### Limitations of Current Approaches

Although the approaches described above have led to important advancements in HIV, HCV and substance use research, there are opportunities to refine how relevant neighborhood exposures are defined to reduce the potential for exposure misclassification. For example, spatial analyses typically use residential addresses to identify hot spots and health service deserts and to calculate distances to services. Similarly, analyses that focus on characterizing the risk environment join neighborhood-level data to individual-level data using one’s place of residence and treat exposure to neighborhood factors as static, rather than dynamic [9,24-27]. Using one’s residential address for these analyses assumes that individuals are only (or are primarily) influenced by their residential environment and that individuals preferentially seek health care at facilities near their home. However, a study conducted among 400 persons receiving primary HIV medical care in Philadelphia reported that most participants traveled farther than the nearest available source of medical care and nearly half traveled more than 3 miles further [28]. As many people spend significant portions of their day away from home and certain behaviors might be more influenced by the social context in which they occur, there is rationale for exploring alternative approaches for classifying one’s neighborhood exposure. For example, the social environment where people inject drugs may be more likely to influence their injection behaviors than their residential environment (if the two are not the same). Similarly, other health behaviors may be influenced more by one’s weighted neighborhood exposure (ie, influenced by different neighborhood exposures according to the amount of time spent in each) than one’s residential neighborhood exposure. Due to convenience (ie, the number of hours spent at work and the overlap between working hours and the operating hours of most health service providers), the availability of health services in the neighborhood where they work versus live (ie, number of providers, type of health service, quality of care), or greater access to public transportation in the neighborhood where they work, some may preferentially seek care closer to where they work than where they live. Consequently, one’s health service use may be more influenced by the neighborhood attributes associated with one’s place of work than one’s place of residence. As some people spend more time away from home (or at work) than others, it is also possible that the association between one’s residential (or work) environment and health service use may be modified by the amount of time spent at that location. Similarly, the association between one’s injection risk behaviors and the injection risk environment may be modified by the amount of time spent in that environment.

Although the vast majority of studies use residential addresses as a proxy, some researchers ask participants to provide addresses or intersections for additional locations, which are then geocoded with varying success [29-33]. For example, in one study, participants were asked to report the intersections nearest to the locations where participants most often hung out during the day, most often slept at night, and most often used drugs (one response per question) [32]. Although this change to the data collection protocol can result in more contextually relevant measures of one’s risk environment, biases in memory and data entry errors can still influence the amount of missing data and the generalizability of study findings. For example, before data collected in this way can be used for analyses or linked with secondary data sources, addresses and intersections must be geocoded. Data entry errors such as spelling errors, missing or incorrectly specified street names (eg, Rd., Blvd., St.), missing street numbers, incorrectly specified directional values (eg *,* North, South), or nonexistent intersections prevent some locations from being geocoded successfully. Furthermore, software programs used to geocode locations (1) are often unable to find matches for some locations, (2) produce multiple matches for others, and (3) do not have error checking programs to ensure that the geocoded locations are valid. In the studies noted above, authors reported being able to successfully geocode approximately 90% of reported addresses and intersections [29,32]. Others noted that participant concerns related to providing exact addresses for one’s residence and illegal activities may have resulted in more incomplete data for these responses; those with missing information on injection locations were significantly more likely to inject in public places and shooting galleries [31]. Missing data can reduce statistical power; missing data and the inclusion of invalidated geographic locations could induce sampling biases [34].

A few researchers have used Google Maps to eliminate the need for geocoding and to improve the accuracy of the location information collected. For example, in one study, interviewers used Google Maps to obtain and validate (via the Google Maps Street View image) each location provided by respondents and then copied and pasted the latitude and longitude coordinates corresponding with each location entry into a database [35]. One limitation of this approach is that there is still a possibility for data entry errors due to errors in the transfer of coordinates from Google Maps to the database. In another study, respondents used Google Enterprise tools including Google Earth and Google Street View to virtually navigate to and pinpoint each location [36]. The interviewer then asked the participant to zoom in to identify the precise location based on visual anchors and landmarks. Following participant confirmation, the interviewer entered the geocoordinates into the corresponding data entry field in a separate Questionnaire Development System (QDS) interview database (QDS Systems, NOVA Research, Bethesda, USA). The authors acknowledged a significant limitation in this approach due to data entry errors that occurred in the transfer of coordinates from Google Street View to QDS. In a subsequent study, this research group developed a software plug-in to reduce this error by directly transmitting geocoordinate information to QDS [36]. Another research team used a Google Map tool embedded in an Internet survey instrument. This survey displayed a map view of Atlanta (initial zoom set to 1:127,000 or 1 inch representing approximately 2 miles). Although respondents could zoom in as much as needed, the zoom level was not recorded in the database and no street-view image was provided for validation [37]. Because users can more precisely locate places when they zoom in further, the error around each estimate will vary according to the zoom level, which is not recorded.

Although the real-time collection of such data in one’s natural environment (ie, geographic momentary assessments [GMA]) has the potential to increase the ecological validity of the data collected and can be used to provide a more comprehensive understanding of the environmental context of risk and health-seeking behaviors among substance-using populations, sometimes the pace of technological innovation exceeds that of ethical standards and guidance [38]. Several researchers have evaluated the feasibility and acceptability of GMA among substance-using populations [39,40], and others have discussed potential privacy and confidentiality concerns, particularly when the behavioral data collected may be illegal or highly stigmatized [38,41,42]. In-depth interviews with persons who use drugs in Baltimore revealed a preference for collecting location-based information using a Web-based mapping survey approach versus GMA. Participants raised privacy and confidentiality concerns associated with GMA; as a result, many said that they would be unwilling to participate in a GMA study or to comply with study procedures. In fact, some said that they would take measures to prevent sensitive location information from being collected. Concerns raised in qualitative interviews with persons who used drugs in Baltimore suggest that, in this setting, GMA could result in differential study participation or study compliance and location data with questionable accuracy or validity for sensitive behaviors [38,41,42].

### Study Objective: Addressing the Gap

To address the limitations of other data collection tools, we developed an interviewer-administered Web-based survey application that interacts with several Google Maps Application Program Interfaces (APIs) to collect and store geographic coordinates and the amount of time spent in each location in a separate and secure database. Our goal was to develop a user-friendly Web-based survey application that could accurately collect data on participants’ key geographic settings. This paper will (1) describe the Web-based survey application developed to collect contextually relevant location information and the amount of time spent in each location using an interviewer-administered survey, (2) examine the extent to which individuals in our sample engage in different activities in nonoverlapping spaces, and (3) compare different approaches for classifying neighborhood exposure (ie, based on where individuals who were recruited for this study live or sleep, work, inject, socialize, and spend the majority of their time, and based on a weighted average of the Baltimore neighborhoods where the individual reports spending time).

## Methods

### Recruitment

Between May 2015 and March 2017, 565 individuals were recruited for a study on HIV care and prevention in Baltimore, Maryland. Due to interviewer error, only 547 of these individuals completed the mapping survey. Participants were recruited using targeted street outreach (n=277 index participants) and peer referral (n=270 network participants). To be eligible to participate, index participants had to be at least 18 years of age, not currently participating in other intervention studies at the research site, and HIV positive (validated with documentation or OraQuick), and should have a history of drug use (heroin, cocaine, or crack) or use any drug to get high including marijuana (effective May 13, 2015). Eligible index participants also had to report one of the following: (1) no HIV medical care in the past 6 months or having gone more than 6 months without seeing a doctor for HIV care in the past 2 years, (2) missed taking prescribed HIV medications in past 90 days, (3) shared injection equipment in the past 90 days, (4) smoked crack in the past 90 days, or (5) unprotected anal or vaginal sex in the past 90 days, and all of the following: (1) currently lives in Baltimore metro area with no plans to move from the Baltimore metro area in the next year, (2) willing to attend group sessions, and (3) willing to talk to people about HIV. Network participants were eligible if they were at least 18 years of age and had received a peer referral coupon from someone verified as an index participant.

### Ascertainment of Key Variables

Using interviewer-administered questionnaires, participants were asked to report the locations (if applicable) where he or she: (1) was when recruited to participate in this study (n=538), (2) most often lived or slept (n=540), (3) most often injected (n=120), (4) most often worked or earned money (n=163), (5) most often socialized (n=426), and (6) spent the most time (n=534) over the last 6 months. Of note, the location or area where participants spent the most time over the last 6 months was asked to (1) capture instances where participants did not spend a majority of their time in one of the other 5 locations and (2) ascertain whether the location where the participant perceived spending the most time overlapped with any of the other location types reported.

**Figure 1:**
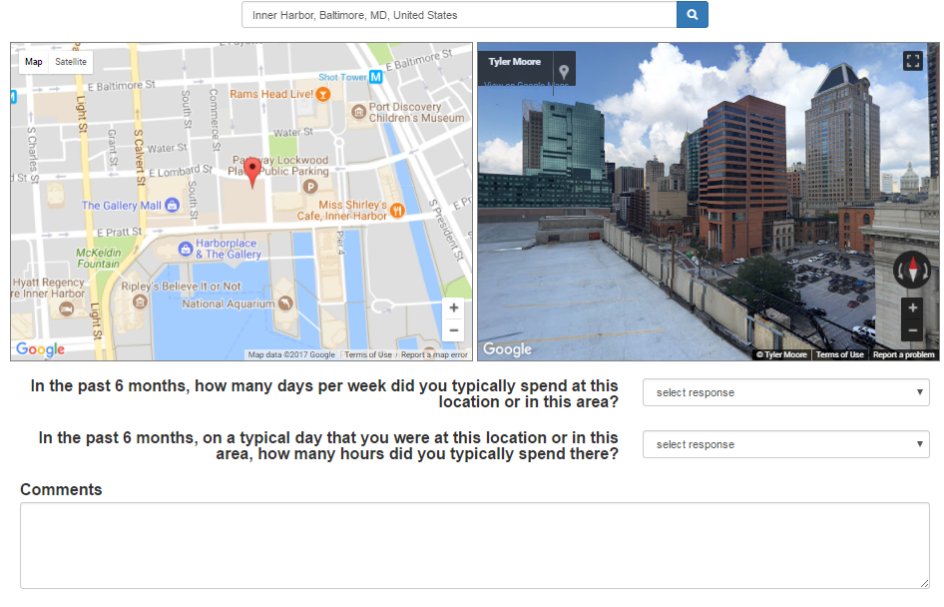
Screenshot of the Web-based map survey.

As seen in the screenshot of this Web-based survey application (Figure 1), each question was presented as a unique map with 2 follow-up questions to ascertain the amount of time spent in the corresponding location. Interviewers first read the location prompt aloud. The participant then provided an address or intersection, which the interviewer entered. If an exact address or intersection was unknown, participants could enter a proxy location or a landmark (ie, church, corner store, park) and then refine their search using the Google Maps navigation features (ie, zoom in and out, rotate, left, right, drag the pointer) to reposition the pointer to the correct location. To prevent instances where participants would otherwise provide misinformation to avoid disclosing certain locations [41,42], interviewers were trained to inform the participant that they could provide a location within a few blocks of the exact location rather than the exact address or use the map to navigate to a location in the correct vicinity that they were comfortable disclosing. After the location was loaded, the participant was asked to confirm whether the location appeared to be correct based on the map-view and the street-view images. If the location was not confirmed by the participant, a new location could be entered without the old location being stored in the database. Only the final location (ie, the corresponding latitude and longitude coordinates; not the location queries) that was confirmed by the participant was stored in the database. Of note, we used the Place Autocomplete feature to provide a type-ahead search box to reduce search errors due to typographical errors in the initial query. After the respondent confirmed the location, the interviewer asked 2 follow-up questions: (1) How many days per week do you typically spend at this location or in this area? and (2) On a typical day that you are at this location or in this area, how many hours do you typically spend there? Response options for the first question include 0-7 days, <1 day, not applicable, and decline to answer. Response options for the second question include 0-24 hours, <1 hour, not applicable, and decline to answer. After selecting responses for each of these questions, the interviewer pushes “submit” to record the coordinates and question responses (but not the location, question details, or address information) in a password-protected MySQL database.

### Web Application Development and Security Features

The Web-based survey application was developed using a MySQL database and the PHP server-side programming language. The program was developed using a model-view-controller framework. The client-side of the application was developed with Hypertext Markup Language, Cascading Style Sheets, and Javascript; it utilizes the Bootstrap Library for user interface elements; the Google Maps API for mapping, street-view, and geocoding functions; and the autocomplete feature of the Places library in Google Maps.

All data (ie, questions, answers, and administrative login information) are stored in a password-protected MySQL database. Of note, although the questions are presented in the survey, the database collects and stores only the unique identifier corresponding with each question and not the question itself. Consequently, there is no label associated with any of the coordinates stored in the database, and the link between the question label and the unique identifier can only be retrieved with an administrative password that is encrypted with a one-way hash. Data can be exported from this administrative view as a .csv file, but only by those with administrative rights.

The resulting database contains only the interviewer identifier, participant identifier, question identifier, coordinates for each question identifier, and the amount of time spent in each location. The website was also protected with a security certificate.

### Statistical Analysis

Of note, because 80.5% (430/534) of participants had not injected drugs in the past 6 months, only 120 participants provided an injection location. Similarly, because the primary source of income for many participants was government-issued assistance or support from network members, only 163 participants provided a location for where he or she worked or earned money. As 83.3% (445/534) of individuals in this sample were unemployed, participants were also permitted to provide the locations for informal sources of income (ie, panhandling, washing cars). Individual-level geographic coordinates (and the corresponding location type and amount of time spent at each set of coordinates) were mapped in ArcGIS 10.2 [43] and assigned to the corresponding Baltimore Community Statistical Area (CSA). In Baltimore City, there are 55 CSAs, 200 census tracts, and over 270 neighborhoods. The Baltimore Neighborhood Indicators Alliance [44] is a repository for Baltimore geographic data, which uses CSAs to present data from multiple sources in a consistent way over time. CSAs were initially designated by the Baltimore Data Collaborative with the Baltimore City Department of Planning according to the following guidelines: CSA boundaries must (1) align with census tracts, (2) consist of 1-8 tracts with 5000 to 20,000 residents, (3) define a demographically homogeneous area, and (4) reflect the city planners’ understanding of residents’ and institutions’ perceptions of community boundaries [44]. This resulted in the loss of 13 individuals, who reported locations outside of this area (N=534 overall, with 118 injection locations and 160 locations reported for where individuals earned money).

To calculate a weighted neighborhood exposure for each participant, we first computed the fraction of time spent in each location by an individual (ie, the amount of time spent in each unique location [numerator] divided by the total amount of time spent in Baltimore [denominator] per person). We then multiplied the fraction of time spent in each CSA by the neighborhood-level data corresponding with that CSA and summed the results for each person. The result is a weighted assessment of one’s neighborhood exposure. In SAS version 9.4 (Cary, NC) [45], we compared the neighborhood exposures by location type (and the weighted neighborhood exposure) using analysis of variance tests with a Bonferroni correction to account for multiple comparisons. For example, given that there are 42 pairwise comparisons for 7 different measures, the adjusted level of significance is 0.05/42, or 0.001.

## Results

### Study Sample

As seen in Table 1, the median age of sampled participants was 51 years (interquartile range [IQR] 43-56), 56.9% (304/534) were male, 62.6% (334/534) had obtained at least a high school degree or the equivalent, 83.3% (445/534) were unemployed, 89.0% (475/534) were black or African American, 85.6% (457/534) had health insurance, and the majority reported using public transportation (78.2% [415/531]), followed by walking (9.6% [51/531]), to get around the city. Of those reporting injection drug use in the past 6 months (19.5% [104/534]), the median time spent traveling to obtain injection drugs was 30 min (IQR 20-60 min).

### Map Survey Descriptive Statistics

The median amount of time required to complete the 6-question map survey was 5 min and 35 s (IQR 4-7 min and 19 s). Participants responded to a median of 4 (IQR 4-5) different location questions (ie, injection and work locations were often not applicable); of these, participants reported a median of 2 (IQR 2-3) different unique locations. As seen in Table 2, locations were considered to be the same if the coordinates matched exactly or were within 0.4 miles (or less than 9 min walking distance from one another). As we permitted individuals to provide approximate addresses for sensitive locations, the distance threshold used to define exact matches in this analysis was informed by our data (ie, locations within several blocks of one another where participants reported spending the same amount of time). The median amount of time spent at home was 89.4%, and the median amount of time spent in the neighborhood where they socialized with friends was 50.0%. Participants spent the least amount of time in the location where they were recruited to participate in this study (median 17.8%). Among those who injected, the median amount of time spent in the location where they injected drugs was 76.1%. Among those who worked, the median amount of time spent in that area was 25.1%.

Overall, the locations where participants reported earning money and being recruited for this study were the least likely to overlap with the locations they reported for other location questions. With respect to overlap in locations, 89.1% (481/540) of the locations where individuals reported living and sleeping overlapped with at least one other location, 81.7% (98/120) of injection locations overlapped with at least one other location, 73.5% (313/426) of locations where individuals reported socializing overlapped with at least one other location, 44.8% (241/538) of locations where participants reported being recruited for the study overlapped with at least one other location, and 41.1% (67/163) of the locations where participants reported working or earning money overlapped with at least one other location (Table 3).

**Table 1:** Sample demographic characteristics, Baltimore, Maryland (N=534), 2014-2017.

Variable		Data
Age, median (IQR^a^)		51 (43-56)
Years in Baltimore (N=533), median (IQR)		47 (34-54)
**Living situation, n (%)**		
	Own house or apartment	267 (50.0)
	With a parent or family member	123 (23.0)
	At someone else’s house or apartment	68 (12.7)
	Rooming, boarding, or halfway house	63 (11.8)
	On the street	5 (0.9)
	Other	8 (1.5)
Homeless in the past 6 months, n (%)		127 (23.8)
**Gender identity, n (%)**		
	Male	304 (56.9)
	Female	221 (41.4)
	Transgender	9 (1.7)
At least a high school diploma or GED^b^, n (%)		334 (62.6)
**Race, n (%)**		
	Black or African American	475 (89.0)
	White	48 (9.0)
	Other or mixed or multiracial	11 (2.1)
Unemployed, n (%)		445 (83.3)
Health insurance, n (%)		457 (85.6)
**Get around city (N=531), n (%)**		
	Drive a car that you own	31 (5.8)
	Drive a car that you borrow	13 (2.5)
	A friend or relative drives you	10 (1.9)
	Taxi or sedan	4 (0.8)
	Public transportation	415 (78.2)
	Walk	51 (9.6)
	Other (bike, drive company cab, someone else drives me, motor wheel chair)	7 (1.3)
**Phone usage, n (%)**		
	Own a cell phone (N=416)	381 (91.6)
	Own a smartphone (N=380)	202 (53.2)
	Own a government-issued phone (N=380)	199 (52.4)
Ever used the Internet (N=415), n (%)		246 (59.3)
Ever used Facebook (N=416) n (%)		181 (43.5)
Neighborhood clean (somewhat or very hopeful) (N=414), n (%)		347 (83.8)
Neighborhood crime (somewhat or very hopeful) (N=413), n (%)		311 (75.3)
Baltimore homicides (somewhat or very hopeful) (N=416), n (%)		278 (66.8)
Community association (yes) (N=383), n (%)		244 (63.7)
Neighborhood activities (yes) (N=415), n (%)		199 (48.0)
Vacant housing (more of a problem on your block) (N=414), n (%)		51 (12.3)
Trash in streets (more of a problem on your block) (N=413), n (%)		64 (15.5)
Groups of teenagers (more of a problem on your block) (N=414), n (%)		97 (23.4)
Selling drugs (more of a problem on your block) (N=414), n (%)		118 (28.5)
Robbed or beaten (more of a problem on your block) (N=413), n (%)		54 (13.1)
**History of injection drug use, n (%)**		
	Never	264 (49.4)
	≥6 months ago	166 (31.1)
	Within the past 6 months	104 (19.5)
**Prior drug treatment enrollment, n (%)**		
	Any drug treatment	277 (51.9)
	Detox (N=276)	67 (24.3)
	Methadone maintenance (N=276)	138 (50.0)
	Outpatient (N=275)	114 (41.5)
	Residential (N=275)	64 (23.3)
	Self-help meeting (N=276)	243 (88.0)
Minutes traveled to get injection drugs (N=127), median (IQR)		30 (20-60)

^a^IQR: interquartile range.

^b^GED: General Equivalency Diploma

**Table 2:** Hours and percentage of time spent in a variety of different types of locations (N=547), 2014-2017.

Location type^a^		Percentage of time spent in each location^b^ Median (interquartile range)	Hours in an average week spent in each location Median (interquartile range)
Live or sleep (N=540)		89.4 (67.2-99.4)	112 (70-147)
Inject (N=120)		76.1 (16.1-99.5)	70 (14-132.25)
Spend most time (N=534)		89.6 (65.9-99.4)	112 (70-154)
Work or earn money (N=163)		25.1 (12.9-47.9)	40 (15.5-55.5)
Socialize (N=426)		50.0 (12.9-99.1)	58 (15-126)
Recruited for study (N=538)		17.8 (2.3-94.1)	19 (3-89.25)

^a^Locations were considered to be the same if the latitude and longitude matched exactly or were within 0.4 miles (or less than a 9 min walking distance apart). In this table, 13 individuals are not included in the neighborhood analyses that joined individual data with neighborhood because they were outside of Baltimore City and no neighborhood indicators were available.

^b^Percentages do not sum to 100% due to overlap.

As shown in Table 3, in total, there were 1224 different locations listed by 547 individuals. Of these 1224 locations, 624 (50.98%) were listed only once by individuals (ie, for one location type), 287 (23.45%) were listed for 2 different locations types, 175 (14.30%) were listed for 3 different location types, 100 (8.17%) were listed for 4 different location types, 30 (2.45%) were listed for 5 different location types, and 8 (0.65%) were listed for all 6 location types. The top row displays the number of locations listed in each category that were listed for that location category only. For example, there were 624 locations reported by individuals that did not overlap with any other location listed by that same individual. Of these, 59 were the locations where participants reported living or sleeping most often in the past 6 months. Of all the locations listed by participants as the places where they lived or slept most often in the past 6 months, this represents 10.9% (59/540). Therefore, the majority of these locations (89.1%) overlapped with at least one other location type listed by a participant; 34.8% (188/540) were also listed for one other activity and 1.5% (8/534) overlapped with all 5 other activities listed. As seen in the top row, 297 of the unique locations listed were the locations where people reported being recruited to participate in this study. This corresponds with over half (55.2% [297/538]) of the recruitment locations listed by participants. The remaining locations overlapped with at least one other location reported by the participant. As seen in the bottom row, 8 individuals reported spending time at the same location for all 6 location types. Of note, 6.9% (37/534) of individuals reported spending most of their time in a location other than one of the locations they reported for the other 5 questions.

**Table 3:** Number of times a particular location is listed by a participant in the 6-question location survey for each location type.

Number of times a particular location is listed by a participant in the 6-question location survey^a^	Live or sleep (N=540), n (%)	Inject (N=120), n (%)	Spend most time (N=534), n (%)	Work or earn money (N=163), n (%)	Socialize (N=426), n (%)	Recruited for study (N=538), n (%)	Total (N=1224), n (%)
1	59 (10.9)	22 (18.3)	37 (6.9)	96 (58.9)	113 (26.5)	297 (55.2)	624 (50.98)
2	188 (34.8)	17 (14.2)	203 (38.0)	23 (14.1)	82 (19.2)	61 (11.3)	287 (23.45)
3	158 (29.3)	26 (21.7)	158 (29.6)	16 (9.8)	98 (23.0)	69 (12.8)	175 (14.30)
4	98 (18.1)	25 (20.8)	98 (18.4)	8 (4.9)	95 (22.3)	76 (14.1)	100 (8.17)
5	29 (5.4)	22 (18.3)	30 (5.6)	12 (7.4)	30 (7.0)	27 (5.0)	30 (2.45)
6	8 (1.5)	8 (6.7)	8 (1.5)	8 (4.9)	8 (1.9)	8 (1.5)	8 (0.65)

^a^Locations were considered to be the same if the latitude and longitude matched exactly or were within 0.4 miles (or less than a 9 min walking distance apart). In this table, 13 individuals are not included in the neighborhood analyses that joined individual data with neighborhood because they were outside of Baltimore City and no neighborhood indicators were available.

**Table 4:** Overlap in location types (N=547).

Location types^a^	Live or sleep (N=540), n (%)	Inject (N=120), n (%)	Spend most time (N=534), n (%)	Work or earn money (N=163), n (%)	Socialize (N=426), n (%)	Recruited for study (N=538), n (%)
Live or sleep	—	71 (59.2)	458 (85.8)	35 (21.5)	218 (51.2)	172 (32.0)^b^
Inject	71 (13.1)	—	66 (12.4)	21 (12.9)	68 (16.0)	46 (8.6)
Spend most time	458 (84.8)	66 (55.0)	—	40 (24.5)	237 (55.6)	172 (32.0)
Work or earn money	35 (6.5)	21 (17.5)	40 (7.5)	—	43 (10.1)	28 (5.2)
Socialize	218 (40.4)	68 (56.7)	237 (44.4)	43 (26.4)	—	157 (29.2)
Recruited for study	172 (31.9)	46 (38.3)	172 (32.2)	28 (17.2)	157 (36.9)	—

^a^Locations were considered to be the same if the latitude and longitude matched exactly or were within 0.4 miles (or less than a 9 min walking distance apart). In this table, 13 individuals are not included in the neighborhood analyses that joined individual data with neighborhood.

^b^A few individuals who indicated recruitment locations that overlapped with the location where they lived or slept most often may have been homeless, been recruited for the study by a friend in their own home, or called the study staff to inquire about potential studies for which they may be eligible.

As seen in Table 4, when the location listed as the area where the participant spent most of their time overlapped with another location, it was most likely to overlap with the location where they reported living or sleeping (85.8% [458/534]), followed by the location where they socialized (44.4% [237/534]). When people reported injecting drugs, the location was most likely to overlap with the locations where they reported living and socializing with friends.

Multimedia Appendix 1 provides the definitions and data sources for the neighborhood indicators used in this analysis. It also provides a summary of the statistically significant differences observed between the neighborhood attributes corresponding with each location type (*P*<.001; alpha=.05/42). Multimedia Appendix 2 compares neighborhood-level exposures according to the location used to define the neighborhood. In general, the neighborhoods where people reported going to work or earn money tended to differ from other locations reported in the following ways: (1) a higher median household income and higher educational attainment; (2) lower rates of poverty, unemployment, and families receiving temporary assistance; (3) fewer vacant or abandoned properties and reports for dirty streets and alleys; (4) more nonviolent crimes but fewer shootings and homicides; and (5) a larger proportion of people walk to work, but a smaller proportion of people take public transportation to work. People reported injecting in neighborhoods characterized by (1) a lower median household income; (2) higher rates of poverty, unemployment, and families receiving temporary assistance; (3) more vacant or abandoned properties; (4) the highest rate of dirty streets and alleys reports; and (5) more shootings and narcotics 911 calls. People lived in areas characterized by lower crime rates and incidents of shootings and with a smaller proportion of the population reporting that they walked to work.

## Discussion

### Feasibility of Administering a Web-Based Survey

The Web-based survey application developed for use in this study facilitated data entry in the following ways: (1) the interviewer and participant could search for each location using an interactive map even when exact addresses were not known, (2) using Google Map and Street Views, participants could confirm that each location entered was correct before it was stored in the database, (3) the Google Place Autocomplete feature was used to reduce typographical errors in the initial query, (4) invalid and missing data entries were further minimized because participants could search for nearby landmarks or cross-streets and use the navigation features to identify more precise locations or locations in the correct vicinity that they were willing to disclose, and (5) each location was automatically geocoded, which reduced data entry errors and missing data.

Using this Web-based survey application permitted the collection of contextually relevant location information and the amount of time spent in each location, both of which can be used to better characterize the risk environment or to better identify health services in close proximity to where participants spend time. Our findings also demonstrate the feasibility of using this Web-based survey application among a sample of persons with a history of drug use and their peer-referrals, who were predominately unemployed (83.3% [445/534]), who reported some homelessness in the past 6 months (23.8% [127/534]), and who did not have much prior experience with the Internet or Google Maps (ie, 40.7% [169/415] reported never having used the Internet).

### Different Neighborhood Exposures by Location Type

To date, most analyses that have examined the influence of neighborhood characteristics on health service use have assumed that individuals are only influenced by the environments in which they live. Our Web-based survey application was developed to address existing methodological limitations. Our analysis demonstrates that participants spent time in multiple nonoverlapping locations and revealed statistically significant differences in one’s environmental exposure when exposure was defined using different areas where participants reported spending time. Our analysis also showed that among those who worked, participants worked and lived in very different types of neighborhoods. For example, the neighborhoods where participants worked were characterized by variables indicating less physical and social disorder compared with all other locations considered. Furthermore, the neighborhoods where participants reported injecting and being recruited for this study had scores indicating higher levels of physical and social disorder.

### Limitations

In this analysis, neighborhood exposures were defined by Baltimore CSAs. Of note, there are 55 CSAs within Baltimore City and 54 are represented in our sample. As there are over 270 neighborhoods in Baltimore, several neighborhoods were combined to compute the indicators for this analysis. Although aggregating individuals to smaller area units could make neighborhood differences more apparent, some measures used in this analysis were not available at more granular levels. Given that our analysis showed significant differences in neighborhood indicators at the CSA level, we would expect to see more differences when neighborhood indicators are incorporated for smaller area units. Future analyses could examine demographic differences between block groups or differences in the neighborhood indicators available for the 278 neighborhoods that comprise the neighborhood inventory for environmental typology (NIfETy) [46]. Of note, 173 of the 278 NIfETY neighborhoods are represented in our dataset. There are also countless other data sources (ie, pollution, crime data, overdose event data, arrest data from the sheriff’s office) that could be merged with these data to examine a myriad of health outcomes. Although using the Google Maps API for mapping, street-view, and geocoding functions, and the autocomplete feature of the Places library in Google Maps facilitated data entry, participants’ responses may still be influenced by recall bias. Additionally, this analysis collected only the location where individuals reported injecting most often, socializing most often, and working most often. Future analyses could collect more detailed information about each unique location within a specific type of location.

### Conclusions

In this manuscript, we show that using an interviewer-administered Web-based geographic data collection approach is feasible among a sample of persons who use drugs in Baltimore, Maryland. In this sample, about half of the locations reported by participants were reported for more than one activity; recruitment locations and locations where people reported going to work or earn money were the least likely to overlap with other location types. Our analyses also show that there were statistically significant differences in the neighborhood environments associated with each of the location types examined (ie, live or sleep, work, socialize, inject, recruit, weighted exposure). Future analyses are needed to (1) determine which neighborhood exposure measures are most strongly correlated with risk and health-seeking behaviors, (2) examine whether the association between one’s environment and health-related outcomes (or risk behaviors) is modified by the amount of time spent in that environment, and (3) compare the availability of health service providers in close proximity to work environments versus other locations (ie, one’s residence). Future research in this and other disciplines could extend these methods to collect the locations where individuals drink alcohol or meet sex partners, and permit multiple locations (and the corresponding amount of time spent in each location) for each location type (ie, multiple injecting locations).

## Appendix

Multimedia Appendix 1 Description of variables and significant findings.

Multimedia Appendix 2 Comparison of neighborhood-level variables by the location used to define the neighborhood.

## Abbreviations

API: application program interface
CSA: community statistical area
GED: General Equivalency Diploma
GMA: geographic momentary assessments
HCV: hepatitis C virus
IQR: interquartile range
NIfETY: Neighborhood Inventory for Environmental Typology
QDS: Questionnaire Development System
