# Supplementary material for: Web-Based Survey Application to Collect Contextually Relevant Geographic Data With Exposure Times: Application Development and Feasibility Testing
Source: JMIR Public Health Surveill. 2018 Jan 19;4(1):e12. doi: 10.2196/publichealth.8581 (PMC5797287; doi:10.2196/publichealth.8581)
Supplement: Multimedia Appendix 1 [file publichealth_v4i1e12_app1.pdf]

Multimedia Appendix 1. Description of variables and significant findings (N=534).

Variable	Definition	Source	Significant differences—Results from analysis of variance with Bonferroni correction (*P*<.001)
Median household income	The median household income is the middle value of the incomes earned in the prior year by households within an area. Income and earnings are inflation-adjusted for the last year of the 5-year period	American Community Survey, 2015	People earned money in areas with significantly higher median household incomes compared with all other neighborhood exposures considered
			People were recruited in areas characterized by significantly lower median household incomes than the weighted neighborhood exposure and the areas where they socialized, spent most of their time, earned money, and lived or slept
Percentage of population (25 years and over) with less than a high school diploma or General Equivalency Diploma (GED)	The percentage of persons who have completed, graduated, or received a high school diploma or GED and also has taken some college courses or completed their associate's degree. This is a standard indicator used to measure the portion of the population with a basic level of skills needed for the workplace. Persons under the age of 25 years are not included in this analysis as many of these persons are still attending various levels of schooling	American Community Survey, 2015	People earned money in neighborhoods with significantly fewer individuals without a high school diploma or GED compared with all other neighborhood exposures considered
Percentage of family households living below the poverty line	The percentage of households whose income fell below the poverty threshold out of all households in an area. Federal and state governments use such estimates to allocate funds to local communities. Local communities use these estimates to identify the number of individuals or families eligible for various programs	American Community Survey, 2015	People earned money in areas with significantly fewer families living below the poverty line compared with all other neighborhood exposures considered
			People were recruited in neighborhoods with significantly more families living below the poverty line compared with the weighted

			neighborhood exposure and the areas where participants socialized and earned money.<br><br>Note: Compared with the neighborhoods where people engaged in other activities, the neighborhoods where people reported being recruited had the highest percentage of families living below the poverty line
Percentage of residential properties that are vacant and abandoned	The percentage of residential properties that have been classified as being vacant and abandoned by the Baltimore City Department of Housing out of all properties. Properties are classified as being vacant and abandoned if the property is not habitable and appears boarded up or open to the elements; the property was designated as being vacant before the current year and still remains vacant; and the property is a multifamily structure where all units are considered to be vacant	Baltimore City Department of Housing	People injected in neighborhoods with significantly more vacant and abandoned properties compared with the weighted neighborhood exposure and the neighborhoods where they reported living or sleeping, spending most of their time, and earning money
			People were recruited in neighborhoods with significantly more vacant and abandoned properties compared with the weighted neighborhood exposure and the neighborhoods where they reported earning money
			People earned money in neighborhoods with significantly fewer vacant and abandoned properties compared with all other neighborhood exposures considered
Percentage of families receiving Temporary Assistance for Needy Families (TANF)	TANF is a federal assistance program. The act provides temporary financial assistance while aiming to get people off of that assistance, primarily through employment	Maryland Department of Human Resources	People injected in neighborhoods with significantly more families receiving TANF compared with neighborhoods where they earned money
			People were recruited in neighborhoods with significantly more families receiving TANF compared with the weighted neighborhood exposure and the neighborhoods where they earned money
			People earned money in neighborhoods with significantly fewer families receiving TANF

			compared with all other neighborhood exposures considered
Unemployment rate	The number of persons between the ages of 16 and 64 years who are in the labor force (and are looking for work) but are not currently working	American Community Survey, 2015	People earned money in neighborhoods with significantly lower unemployment rates compared with all other neighborhood exposures considered
Rate of dirty streets and alleys reports per 1000 residents	The rate of service requests for dirty streets and alleys through Baltimore	Baltimore City CitiStat	People earned money in neighborhoods with significantly lower rates of dirty streets and alleys reports compared with the locations where participants reported injecting, socializing, spending most of their time, and living or sleeping
			People injected in neighborhoods with the highest rate of dirty streets and alleys reports; however, the only significant difference was with where people earned money
Violent crimes per 1000 residents	Incidents of homicide, rape, aggravated assault, robbery, burglary, larceny, and auto theft that are reported to the Police Department per 1000 residents	Baltimore City Police Department, 2015; US Census, 2015	People were recruited in neighborhoods with significantly higher rates of violent crime compared with the weighted neighborhood exposure and the neighborhoods where they reported injecting, spending most of their time, and living or sleeping
			People socialized in neighborhoods with significantly higher rates of violent crime compared with the neighborhoods where they spent most of their time and lived or slept
Property crime rate per 1000 residents	The number of property-based crimes (burglary and auto theft) that are reported to the Police Department per 1000 residents	Baltimore City Police Department, 2015; US Census, 2015	People earned money in neighborhoods with significantly higher rates of property crime compared with the weighted neighborhood exposure and the neighborhoods where people reported spending most of their time, injecting, and living or sleeping
			People were recruited in neighborhoods with significantly higher rates of property crime compared with the weighted neighborhood exposure and the neighborhoods where they reported injecting, spending most of their time, and living or sleeping
			People socialized in neighborhoods with significantly higher rates of property crime compared with the neighborhoods where they spent most of their time and lived or slept

Number of shootings per 1000 residents	The rate of 911 calls for shootings per 1000 residents in an area. Because the data come from 911 calls, it is possible that multiple calls could be made for a single incident	Baltimore City Police Department, 2015; US Census, 2015	People were recruited in neighborhoods with significantly more shootings compared with the weighted neighborhood exposure and the neighborhoods where they reported spending most of their time, socializing, earning money, and living or sleeping
			People injected in neighborhoods with significantly more shootings compared with the neighborhoods where they earned money
			People socialized in neighborhoods with significantly more shootings compared with the neighborhoods where they earned money
Number of gun-related homicides per 1000 residents	The number of homicides by firearm reported per 1000 residents in an area	Baltimore City Police Department, 2015; US Census, 2015	People earned money in neighborhoods with significantly lower rates of gun-related homicides compared with all other neighborhood exposures considered
Number of common assault calls for service per 1000 residents	The number of calls for assaults that do not involve a weapon per 1000 residents in an area. Because the data come from 911 calls, it is possible that multiple calls could be made for a single incident	Baltimore City Police Department, 2015; US Census, 2015	People were recruited in neighborhoods with significantly more 911 calls for common assaults compared with the weighted neighborhood exposure and the neighborhoods where they reported spending most of their time, injecting, and living or sleeping
			People socialized in neighborhoods with significantly more 911 calls for common assaults compared with the neighborhoods where they spent most of their time and lived or slept
Number of narcotics calls for service per 1000 residents	The number of calls for narcotics per 1000 residents in an area. Because the data come from 911 calls, it is possible that multiple calls could be made for a single incident	Baltimore City Police Department, 2015; US Census, 2015	People injected in neighborhoods with significantly more narcotics 911 calls compared with the neighborhoods where they earned money
Number of adult arrests per 1000 residents (over the age of 18 years)	The number of persons aged 18 years and over arrested per 1000 adults who live in an area. This indicator is calculated by where the individual was arrested and not by where the crime is committed	Baltimore City Police Department, 2015; US Census, 2015	People were recruited in neighborhoods with significantly more adult arrests compared with the weighted neighborhood exposure and the neighborhoods where they reported spending most of their time and living or sleeping
Percentage of households with no vehicles available	The percentage of households that do not have a personal vehicle available for use out of all households in an area	American Community Survey, 2015	People were recruited in neighborhoods with significantly more households without a vehicle compared with the weighted neighborhood exposure and the neighborhoods

			where they socialized, spent most of their time, lived or slept, and earned money
			People earned money in neighborhoods with significantly fewer households without a vehicle compared with neighborhoods associated with all other locations considered
Percentage of population that use public transportation to get to work	Percentage of commuters that use public transit out of all commuters aged 16 years and above	American Community Survey, 2015	People earned money in neighborhoods with a significantly smaller proportion of the population using public transportation to go to work compared with the neighborhoods associated with all other locations considered
			People were recruited in neighborhoods with a significantly larger proportion of the population using public transportation to go to work compared with the weighted neighborhood exposure and neighborhoods where people earned money
Percentage of population that walks to work	Percentage of commuters who walk to work out of all commuters aged 16 years and above	American Community Survey, 2015	People were recruited in neighborhoods with a significantly larger proportion of the population that walks to work compared with the weighted neighborhood exposure and the neighborhoods associated with the locations where participants socialized, injected, spent most of their time, and lived or slept
			People earned money in neighborhoods with a significantly larger proportion of the population that walks to work compared with neighborhoods where participants spent most of their time and lived or slept
			People socialized in neighborhoods with a significantly larger proportion of the population that walks to work compared with neighborhoods where participants spent most of their time and lived or slept
