# Supplementary material for: Web-Based Survey Application to Collect Contextually Relevant Geographic Data With Exposure Times: Application Development and Feasibility Testing
Source: JMIR Public Health Surveill. 2018 Jan 19;4(1):e12. doi: 10.2196/publichealth.8581 (PMC5797287; doi:10.2196/publichealth.8581)
Supplement: Multimedia Appendix 2 [file publichealth_v4i1e12_app2.pdf]

Multimedia Appendix 2. Comparison of neighborhood-level variables by the location used to define the neighborhood.

Variable	Live or sleep (n=495) Median (IQR[a])	Inject (n=113) Median (IQR)	Spend most time (n=491) Median (IQR)	Work or earn money (n=134) Median (IQR)	Socialize (n=399) Median (IQR)	Recruited (n=515) Median (IQR)	Weighted (n=534) Median (IQR)
Median household income	US $31,759 (US $25,152-US $38,174)	US $29,364 (US $23,626-US $34,879)	US $31,759 (US $25,152-US $38,683)	US $38,174 (US $29,364-US $46,133)	US $31,759 (US $24,108-US $39,300)	US $29,364 (US $18,755-US $38,174)	US $32,214 (US $26,136-US $38,440)
Percentage of population (25 years and over) with less than a high school diploma or GED[b]	22 (16.7-31.4)	24.6 (20.4-31.4)	22 (16.7-31.4)	18.85 (10.5-25.3)	22 (15.8-30.9)	23.1 (15.8-32.6)	22 (17.0-27.9)
Percentage of family households living below the poverty line	25.8 (19.8-33)	26.3 (23.6-35.8)	25.8 (19.5-33)	23.6 (8.7-26.3)	25.8 (18.6-33)	26.3 (21.3-44.4)	25.6 (20.2-33.0)
Percentage of residential properties that are vacant and abandoned	16.4 (3.6-26.6)	19.9 (5.4-28.1)	16.4 (3.6-26.6)	5.4 (2.7-19.9)	16.4 (5-26.7)	19.9 (5.4-26.6)	15.2 (5.3-24.8)
Percentage of families receiving Temporary Assistance for Needy Families (TANF)	13.5 (7.9-19.4)	16 (9.5-21.3)	13.5 (7.7-19.4)	8.65 (5.2-15)	14.5 (7-19.8)	16 (7-19.8)	13.5 (7.9-18.9)
Unemployment rate	15.1 (11.9-18.3)	15.1 (13.8-18.4)	14.8 (10.8-18.3)	14.25 (5.9-15.7)	14.8 (8.2-18.4)	14.8 (8.2-16.6)	14.8 (11.5-17.3)
Rate of dirty streets and alleys reports per 1000 residents	79.3 (49-168.1)	86.1 (58.1-168.1)	79.3 (49-168.1)	57.6 (44.3-110)	78.2 (53.4-168.1)	68.2 (44.3-168.1)	80.6 (52.3-147.4)
Violent crimes per 1000 residents	21.4 (15.8-26.5)	23.5 (19-27.5)	21.4 (15.8-26.5)	21.4 (15.5-28.3)	23.5 (18.8-27.6)	26.5 (20.9-29.9)	22.6 (17.5-26.7)

Property crime rate per 1000 residents	49.2 (41.2-64.2)	55.3 (45-66.2)	49.2 (41.2-66.2)	55.3 (45-67)	55.3 (45-66.7)	55.5 (47-66.2)	54.2 (43.9-66.0)
Number of shootings per 1000 residents	6.1 (2.4-7.5)	6.8 (2.6-8.3)	6.1 (2.4-7.5)	2.95 (1.6-7.4)	6.4 (2.4-7.5)	7 (2.6-9.1)	5.7 (2.6-7.5)
Number of gun-related homicides per 1000 residents	0.9 (0.3-1)	1 (0.3-1.1)	0.9 (0.3-1)	0.35 (0-1)	0.8 (0.2-1)	1 (0.2-1)	0.8 (0.3-1.0)
Number of common assault calls for service per 1000 residents	82.3 (66.5-109.6)	101.3 (75.4-109.6)	88 (66.5-114.4)	85 (63.8-116.1)	101.3 (74.2-116.1)	102.8 (74.2-139)	92.4 (71.8-114.4)
Number of narcotics calls for service per 1000 residents	78.8 (33.4-141)	108.3 (56.8-168.1)	78.8 (33.4-141)	48.9 (24.9-136.5)	81.5 (33.4-144.8)	73 (42.1-141)	78.8 (41.4-141.0)
Number of adult arrests per 1000 residents (over the age of 18 years)	49.1 (23.2-70)	57.8 (29.3-70)	51.7 (23.2-70)	35.7 (16.7-70)	57.8 (23.2-70)	57.8 (26.5-70)	49.1 (26.5-68.5)
Percentage of households with no vehicles available	43.2 (30.4-55.2)	43.4 (42-57)	43.2 (31.6-55.2)	39.8 (29.1-43.7)	43.2 (36.1-55.2)	43.4 (40.1-57.3)	42.9 (33.6-53.1)
Percentage of population that use public transportation to get to work	29.3 (20.6-33.6)	32 (23-34.8)	30.1 (20.6-33.6)	21.8 (14.3-31.3)	30.1 (20.6-33.7)	31.3 (20.6-34.8)	28. 1 (20.6-33.0)
Percentage of population that walks to work	6.4 (3.8-13.4)	8.5 (4.3-13.4)	7.2 (4-13.4)	8.7 (4.2-18.3)	8.6 (4.3-17.9)	13.4 (6.4-22.7)	8.1 (4.4-13.5)

[a]IQR: interquartile range.

[b]GED: General Equivalency Diploma.
